# Finding a Universal Execution Strategy for Model Transformation Networks

**DOI:** 10.1007/978-3-030-71500-7_5

**Published:** 2021-02-24

**Authors:** Joshua Gleitze, Heiko Klare, Erik Burger

**Affiliations:** 8 Universidad Autónoma de Madrid, Madrid, Spain; 9 University of Twente, Enschede, The Netherlands; KASTEL, Karlsruhe Institute of Technology, Karlsruhe, Germany

**Keywords:** model consistency, model transformation networks

## Abstract

When using multiple models to describe a (software) system, one can use a network of model transformations to keep the models consistent after changes. No strategy exists, however, to orchestrate the execution of transformations if the network has an arbitrary topology. In this paper, we analyse how often and in which order transformations need to be executed. We argue why linear execution bounds are too restrictive to be useful in practice and prove that there is no upper bound for the number of necessary executions. To avoid non-termination, we propose a conservative strategy that makes execution failures easier to understand. These insights help developers and users of transformation networks to understand under which circumstances their networks can terminate. Additionally, the proposed strategy helps them to find the cause when a network cannot restore consistency.

## 1 Introduction

When modelling systems, one is often confronted with the task of *model consistency*: Since model-driven development aims at separating concerns by tailoring models to the needs of the people working on the system, there are typically different models, each one capturing the parts of the system that are relevant to the model's target audience. All those models taken together should describe a coherent system and not contain contradictory information. We say that the models should be consistent. Automatic detection and resolution of inconsistencies is, however, still poorly addressed in current development processes [12].

There are different means of maintaining consistency. A popular one is to define *incremental model transformations*, which update models based on information that was changed in one of them. While there has been significant research on model transformations themselves, particularly on binary transformations, maintaining consistency of multiple models is less researched [2]. There are approaches for multiary model transformations which can transform between multiple models by means of a single transformation. Nevertheless, one will likely

* This work was supported by funding of the Helmholtz Association (HGF) through the Competence Center for Applied Security Technology (KASTEL).

also want to be able to combine multiple transformations—binary or multiary—to maintain consistency, creating a *transformation network*. Unlike using a single, overarching transformation, defining a network makes it possible to reuse modular ones. Additionally, knowledge about consistency between certain types of models is often distributed across domain experts [13]. This can be accommodated by transformation networks, because every domain expert can define transformations independently and according to their view on consistency.

To the best of the authors' knowledge, no strategy that determines an execution order of transformations to maintain consistency in a network with arbitrary topology has been presented yet. Existing work proposes, for example, defining an execution order explicitly [23, 35] or deriving a topological order [30]. Most approaches restrict the supported kinds of network topologies to such in which each transformation only needs to be executed once.

In this paper, we research properties and limitations of a universal strategy that executes a transformation network of arbitrary topology. We show that strategies that apply each transformation only once are not useful in practice. At the other end of the spectrum, we prove that not limiting the number of transformation executions does, in general, lead to non-termination. Based on the insight that a universal strategy can only operate conservatively, we derive a practicable strategy. In detail, we make the following contributions:

**Formalisation *(C1)*:** We formalise transformation networks and execution strategies to precisely define their expected properties.
**Conservativeness Proof *(C2)*:** We prove that a universal execution strategy must operate conservatively to avoid non-termination.
**Strategy Design *(C3)*:** We propose a strategy that improves explainability whenever no consistent models are found.

The contributions establish fundamental knowledge about the design space of network execution strategies, their undecidability, and difficulties in reducing conservativeness. The proposed strategy helps transformation network developers and users to find the reasons when an execution does not yield consistent models.

## 2 Problem Statement

In this section, we will further motivate our research by giving an example and clarifying its context. We provide a formalisation for transformation networks and execution strategies to generate a common understanding and formal basis for transformation network orchestration, constituting contribution *C1*.

### 2.1 Motivating Example

Figure 1 depicts a software project whose contributors take the roles of architects, developers and user experience (UX) designers. One person can take multiple roles, but every role has a particular view on the project and uses related tools. Architects use a UML-based tool to analyse and plan the architecture. Developers

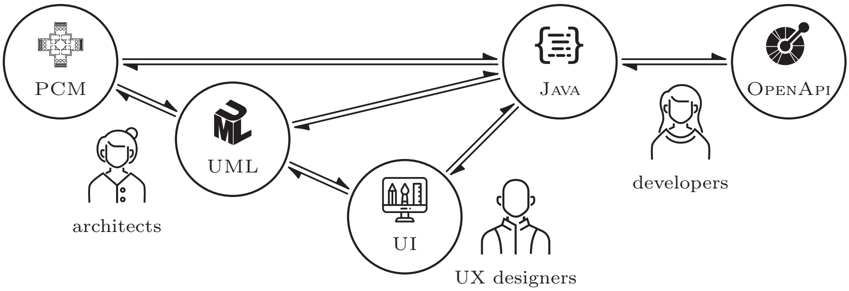

**Fig. 1.** Example for a transformation network in model-driven (software) development.

program the software in Java. These two models overlap: Although they cannot be derived completely from each other, the implementation should follow the architecture and architects want to see how code changes affect the architecture.

UX designers develop the UI for the software. Their designs overlap with the UML model, because, first, the software's requirements mandate certain properties of the UI, and, second, the architecture may restrict which information can be shown at which point in the interface. The UI design also overlaps with the code, since static parts of the UI can be derived from the UI model. Ideally, changes in the UI code can even be propagated back into the UI model.

The developers use OpenAPI™ [32] to exchange specifications of HTTP APIs. These specifications overlap with the parsing and serialisation code. Architects want to analyse how their architecture choices influence performance, using the Palladio Component Model (PCM) [24]. The architecture specification used in the PCM overlaps with the one defined in UML. Additionally, the PCM model contains information about performance properties and the deployment structure, which can partially be derived from the code.

Those relations can be encoded in transformations to avoid re-specification of similar information, such as the architecture in PCM and UML, to derive information, like appropriate Java stubs from OpenAPI specifications, and to preserve information consistency. Figure 1 shows the resulting transformation network. In this paper, we will find an execution strategy for such transformations, which is needed to correctly propagate changes from one model to the others.

### 2.2 Context

We discuss model transformation networks in a specific usage context. We assume that different roles are involved in a development project, each using some models to describe their view of the system. The models are kept consistent by model transformations. For the sake of simplicity, we only discuss *binary* transformations between two models. To foster independent specification and reuse of transformations, we assume that they are not tailor-made, but may be general-purpose. As a consequence, we cannot assume that the models or transformations are or can be aligned, for example, to ensure that their execution in a specific order always results in consistent models. Neither can we assume that the network has a certain topology. We do, however, assume that all transformations are in accordance to a well-defined overall notion of consistency (reaching a consistent state would be impossible otherwise). This means that all requirements we pose on the transformations must only concern a transformation itself. A requirement like "no transformation overwrites the result of another" would not fit our context.

We require that transformations are *synchronising* [4], i.e., that they can deal with the situation that both of their models have been changed. This is essential to find an execution strategy: When propagating changes in a transformation network that contains cycles, it will inevitably happen that both models that are connected by a transformation will be changed. In addition, the well-researched *bidirectional* transformations only change one of the models [28] and could in such a situation be forced to overwrite changes to yield a consistent result. This assumption also enables concurrent modifications by different project members.

### 2.3 Formalisation

We are not concerned with how models are structured, so we simply resort to defining a universe $\mathbb{M}$ that contains all models. First, we define the kind of transformations that we use:

**Definition 1.** *A* synchronising binary transformation (syncx) $\vec{t}$ *is a function that updates two models:*

$$\vec{t}\colon (\mathbb{M} \times \mathbb{M}) \to (\mathbb{M} \times \mathbb{M})$$

*A syncx' image consists of fixed points:*

$$\forall a \in \mathbb{M}\, \forall b \in \mathbb{M} : \vec{t}\big(\vec{t}(a,b)\big) = \vec{t}(a,b)$$

*The universe of all syncx for* $\mathbb{M}$ *is called* $\mathbb{T}$*.*

This formalisation is a simplification sufficient for the purposes of this paper. In practice, transformations will, for example, be allowed to indicate an error instead of being required to always produce appropriate new models.

In comparison to existing formalisms [28], there is no consistency relation in the definition of a syncx. For our purposes, the consistency relation is not part of a syncx, but rather encoded implicitly in the syncx' behaviour. We assume that the transformations are correct and hippocratic [28] with regard to their implicit consistency relation and can then recover the relation:

**Definition 2.** *The* consistency relation $R_{\vec{t}}$ *of syncx* $\vec{t}$ *is given by:*

$$R_{\vec{t}} = \big\{(a,b) \mid \vec{t}(a,b) = (a,b)\big\}$$

This paper focuses on transformation networks that are created when combining multiple syncx:

**Definition 3.** *A* transformation network $N =: ((V,E),T)$ *consists of a directed, connected, self-loop-free graph* $G = (V,E)$ *and a syncx assignment* $T\colon E \to \mathbb{T}$*. Any two vertices* $\{a,b\} \subseteq V$ *have at most one edge between them:* $(a,b) \in E \implies (b,a) \notin E$*. The universe of all model transformation networks for* $\mathbb{M}$ *is called* $\mathbb{U}$*.*

A transformation network captures the topology and the used transformations. There is no inherent reason to exclude multigraphs or self-loops. We use this simpler definition because it makes it easier to argue about the networks without restricting expressiveness. We use directed edges instead of undirected ones to provide a notion of the “left” and “right” model for a syncx. The edges’ direction does not indicate anything about the direction of change propagation. We will usually regard the network as given and try to find suitable model assignments:

**Definition 4.** *For a transformation network $N =: ((V, E), T)$, a* model assignment *$M$ is a function $M : V \to \mathbb{M}$.*

Naturally, we are particularly interested in model assignments that are consistent with the transformations:

**Definition 5.** *For a transformation network $N =: ((V, E), T)$, a model assignment $M$ is* consistent *if, and only if*

$$\forall (a, b) \in E : (M(a), M(b)) \in R_{T(a,b)}$$

*The set of all consistent model assignments for $N$ is called $R_N$.*

We use the following additional notation in this paper:

- “$A \to B$” for the set of functions from set $A$ to set $B$
- “f: $A \nrightarrow B$” for a partial function $f$ from $A$ to $B$
- “$f(x) = \bot$” to mean that a partial function $f$ is not defined at $x$
- “Im($f$)” to denote the image of a function $f$

### 2.4 Problem Description

Our goal is to find an algorithm that, given a transformation network $N =: ((V, E), T) \in \mathbb{U}$ and a model assignment $M$, finds a consistent model assignment $M'$ by applying transformations in $\mathrm{Im}(T)$. We call such an algorithm a “(transformation network) execution strategy”. It is “universal” if it is parametrised by and thus defined for every network.

**Definition 6.** *A universal execution strategy determines an order (i.e., a permutation with duplicates) of transformations in $\mathrm{Im}(T)$ for a given transformation network $N =: ((V, E), T) \in \mathbb{U}$ and model assignment $M \in (V \to \mathbb{M})$. It realises a partial function $S : \mathbb{U} \times (V \to \mathbb{M}) \nrightarrow (V \to \mathbb{M})$.*

An execution strategy finds a new model assignment only by executing the transformations of the network, as more precisely defined by Klare et al. [15, Definition 8]. If $S(N, M) \neq \bot$, we say that the strategy “resolves” $N$ and $M$. If $S(N, M) = \bot$, we say that the strategy fails. We have further requirements:

**Requirement 1.** *An execution strategy must be correct:*

$$\forall N =: ((V, E), T) \in \mathbb{U} \;\; \forall M \in (V \to \mathbb{M}) : S(N, M) \in R_N \cup \{\bot\}$$

**Requirement 2.** *An execution strategy must be hippocratic:*

$$\forall N =: ((V, E), T) \in \mathbb{U} \;\; \forall M_c \in R_N : S(N, M_c) = M_c$$

An execution strategy will not always be able to find a consistent new model assignment (i.e., there will be some $N, M$ such that $S(N, M) = \bot$). First, there may not be a consistent model assignment at all (i.e., $R_N = \emptyset$). Second, there may be a consistent model assignment but no execution order of the transformations that yields that assignment [30, 16]. We call such inputs "unresolvable" [30]. Conversely, if there is an execution order of the transformations that yields a consistent model assignment, we call the inputs "resolvable".

An execution strategy may even fail for resolvable inputs: The execution strategy may not "find" a consistent model assignment, even though it is reachable. For example, the strategy may abort before having executed the transformations often enough, or finding the assignment might require an order of execution which the strategy does not consider. We call such a strategy "conservative":

**Definition 7.** *An execution strategy $S$ is* conservative *if it is correct and if there can be resolvable inputs $N, M$ with $S(N, M) = \bot$.*

The higher the probability that an execution strategy yields a result for resolvable inputs (we also say the lower its "level of conservativeness"), the more useful the strategy will be. It is, however, also desirable that the strategy is predictable, meaning that one can determine beforehand for which inputs the strategy will succeed. For example, it would be useful to know whether a strategy yields a result for a given network for *any* resolvable model assignment. Informally speaking, we would like to have an "easy-to-check" criterion for transformation networks determining whether this is the case. An even better criterion could be applied to a single syncx, such that the strategy can resolve all inputs with a network of syncx that fulfil the criterion. This would be ideal for the motivated context of independently developing and freely combining syncx to a network.

To summarise, we aim to find a correct, hippocratic execution strategy that is able to keep models consistent via transformation networks. The strategy should succeed for realistic inputs with a high probability. Additionally, we aim to find criteria that determine the cases in which the strategy will succeed.

## 3 Related Work

Approaches for restoring model consistency have been subject to intensive research, surveyed by Macedo et al. [21]. Model transformations are a well-researched option, and several tools and languages have been developed to support them [27, 18, 25]. Research has, however, mainly focused on consistency between two models, which also concerns theoretical properties like *termination* as one of the properties that we investigate for the execution of transformation networks [7]. Maintaining consistency between more than two models has recently gained more attention, especially in terms of a dedicated Dagstuhl seminar [2]. The central approaches of multiary transformations and networks of binary transformations can be distinguished. In Section 1, we have discussed that multiary transformations are complex to specify, whereas networks of binary transformations have limited expressiveness [30], which does, however, not seem to be practically relevant [2].

*Multiary Transformations:* Different approaches for multiary transformations have been proposed. QVT-R [22] supports multidirectionality already by design, but ambiguities in the standard limit practical applicability [20]. Triple Graph Grammars (TGGs) [26] are bidirectional specifications, which are well-suited for model transformations [1]. Extensions of TGGs to multiple models called Multi Graph Grammars (MGGs) [17] and Graph Diagram Grammars [34, 33] consider the specification of multidirectional rules. All these approaches, however, require the transformation developer to know about and be able to express the relations between all involved models, which we reasonably excluded by assumption.

*Auxiliary Models:* Not all multiary relations can be expressed by sets of binary ones. Adding one auxiliary model makes it, however, theoretically possible to express arbitrary multiary relations by binary ones [30]. Some work discussed which kinds of relations can be expressed with such an approach and how they can be formalised in the lenses framework [5, 31]. Other work discussed how composing such auxiliary models to express commonalities of models can be achieved [14]. Such auxiliary models actually encode a multiary transformation in a model together with binary transformations to the models to keep consistent, resulting in the same challenges as for transformation network. In consequence, our work on transformation networks is also required and applicable there.

*Binary Transformations:* Although they cannot express all multiary relations, there are arguments in favour of using networks of modular transformations, especially binary ones: They are easier to develop when domain knowledge is distributed [13] and they are easier to comprehend by a single developer [2, 30]. Additionally, binary transformations are researched well and a variety of tools supporting different kinds of specifying them exist [27, 18, 25, 21]. Most formalisms and tools consider *bidirectional* transformations, whereas networks require synchronising transformations, as motivated in Section 2.2. Non-synchronising transformations can, however, be adapted to become synchronising [37].

*Transformation Chains:* Transformation chains combine transformations to derive low-level models from high-level ones across intermediate representations. Languages like FTG+PM [19] and UniTI [35] enable the specification of such chains. Transformation chains are, however, only a special case of general transformation networks. Etien et al. consider specific properties of transformation chains. They investigate how conflicts in terms of results depending on the execution order can be detected [8]. These results do, however, not aim to relieve developers from the task of finding an execution order manually, as we do in this paper.

*Transformation Composition:* Transformation composition techniques are a means to build networks of binary transformations. They can be separated into internal, white-box approaches [36], and external techniques, which consider transformations as black-boxes. Our contributions can be seen as an external composition technique. However, composition usually considers transformations between the same rather than different types of models. From a theoretical perspective (see Section 2.3) this could be treated equally by not distinguishing models by their metamodels. Practical approaches, however, consider transformations between specific metamodels rather than arbitrary models.

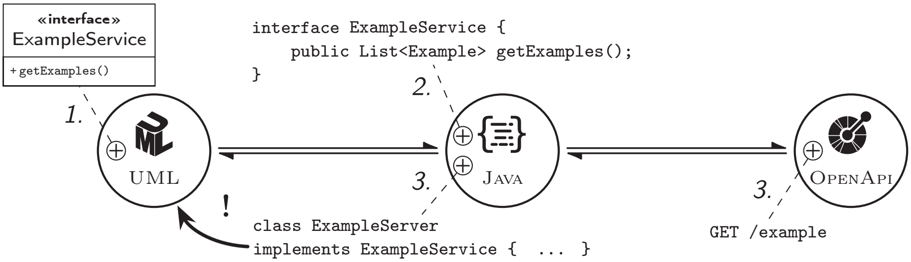

**Fig. 2.** Example yielding inconsistent models after executing each transformation once. Numbers in italics indicate the order in which changes are performed.

*Execution Strategies:* Di Rocco et al. [3] describe a simple strategy for orchestrating transformations, but make strong assumptions requiring that each of them is only applied once. Stevens [30] proposes a strategy that also executes each transformation only once in one direction. It includes a notion of authoritative models, which are not allowed to be changed, and does not consider synchronising transformations. Likewise, Stevens [29] proposes to find an *orientation model* defining in which direction transformations are executed. If, however, several transformations modify the same model, the approach leaves it to the developer to determine an execution order after which all consistency relations hold. Such strategies are only correct if the network is a tree, or if no transformations interfere with each other. We present a simple scenario in which this is already too limiting in Section 4.1. We overcome this limitation by executing transformations more than once and thereby letting them "negotiate" a result even if they interfere, which yields a *universal* execution strategy for arbitrary network topologies.

## 4 Design Space

We approach the possibilities for designing an execution strategy by looking at how often it executes syncx in the worst case. We consider the two extremes of executing every syncx at most once and executing them an unlimited number of times, and find that neither of them will do: While the first one is too limiting, the second one cannot guarantee termination. As a consequential insight, a universal execution strategy needs to be *conservative*, introduced as contribution *C2*.

### 4.1 One Execution per Transformation

Several proposed strategies execute every transformation in a network at most once [30, 35]. Since we expect that transformations are developed independently, and are thus not necessarily aligned (see Section 2.2), restricting the number of executions to one per transformation would, however, limit the possible combinations of them, and models could not be kept consistent in desirable scenarios. We give an example for this in the following.

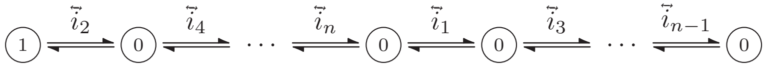

**Fig. 3.** A transformation network with $\boldsymbol{n}$ transformations reacting to each other.

We use the example of Section 2.1, and focus on the UML, Java and OpenAPI models to consider the scenario visualised in Figure 2: An architect creates a new UML interface and applies an execution strategy that executes every transformation once. First, the UML-to-Java syncx creates an appropriate interface in Java. The OpenAPI-to-Java syncx recognises that the interface should be exposed via an HTTP API and creates a matching endpoint in the OpenAPI model. Additionally, it creates a stub implementation with parsing and serialisation code in Java. The stub implementation classes can, however, not be propagated back to UML, because the UML-to-Java syncx has already been executed.

We see that if we limit the number of executions to one per transformation, transformations cannot propagate back the changes that other transformations have made. However, in the context described in Section 2.2, it is necessary that transformations are able to "react" to the changes made by other transformations. This offers, for instance, separation of concerns: The logic for a certain aspect of consistency can be put in only one transformation and other transformations will propagate it throughout the network. Without such a mechanism, all aspects of consistency would need to be implemented in all transformations. This would cause duplication of logic and reduce reusability of transformations, which would be impractical and contradicts our assumption of independent development. If we added the logic for creating implementations of relevant Java interfaces to the UML-to-Java syncx, then it would implicitly assume the presence of the Java-to-OpenAPI syncx. It could, thus, not be easily reused in networks where the Java-to-OpenAPI syncx is not used.

We can generalise the previous example: Let the model universe be the natural numbers: $\mathbb{M} = \mathbb{N}_0$. Let further for any $1 \leq j \leq n$ the syncx $\overleftrightarrow{i}_j$ be defined as

$$\overleftrightarrow{i}_j\colon (a,b) \mapsto \begin{cases} (m+1, m+1) & \text{if } m = j \\ (m,m) & \text{else} \end{cases} \qquad \text{with } m := \max\{a,b\}$$

$\overleftrightarrow{i}_j$ sets both models to the higher number of the two, except if that number is $j$. Then $\overleftrightarrow{i}_j$ increments the result by one. This is an abstraction of syncx "reacting" to each other: The $\overleftrightarrow{i}_j$s seek to set all models to the same value, except that after $\overleftrightarrow{i}_{j-1}$ was executed, $\overleftrightarrow{i}_j$ changes its behaviour and increments the value by one.

We now construct the transformation network $N_n$ for $n = 2k, k \in \mathbb{N}^+$ (see Figure 3) with $n$ indicating the number of syncx within the network, and examine how many executions it requires:

$$T_n = (i, i+1) \mapsto \begin{cases} \overleftrightarrow{i}_{2i} & \text{if } i \leq \frac{n}{2} \\ \overleftrightarrow{i}_{2i-n-1} & \text{else} \end{cases}$$

$$N_n = (([1, n+1], \{(i, i+1) \mid i \in [1, n]\}), T_n)$$

**Lemma 1.** *$\vec{i}_n$ must be executed at least $n$ times to resolve $N_n$ with the initial model assignment*

$$M_1 : i \mapsto \begin{cases} 1 & \text{if } i = 1 \\ 0 & \text{else} \end{cases}$$

*Proof.* The only reachable model assignment that is consistent is $M_n : i \mapsto n$. It is reached by having every $\vec{i}_j$ increment the highest number in the model assignment by one if that highest number currently is $j$. All transformations incrementing even numbers are on one side of $\vec{i}_n$ (except for $\vec{i}_n$ itself), all transformations incrementing uneven numbers are on the other side. Thus, the currently highest number must be propagated to the other side of $\vec{i}_n$ at least $n-1$ times. Additionally, $\vec{i}_n$ must increment $n-1$ to $n$. □

**Theorem 1.** *For any execution strategy that uses $\mathcal{O}(1)$ executions of each transformation, there are inputs that the execution strategy cannot resolve.*

*Proof.* Follows directly from Lemma 1. □

The example network in Figure 2 is a simplification of a realistic transformation scenario, which we generalised to the network $N_n$. In consequence of Theorem 1, we can expect that transformation networks can, in general, not be resolved with $\mathcal{O}(1)$ executions of each transformation.

### 4.2 Unlimited Executions

We now consider an execution strategy that executes transformations as long as they still change models, and terminates once no more changes occur. This overcomes the shortcoming that we observed with limiting the number of executions to a constant; we will, however, see that we cannot guarantee termination of such an execution strategy. By simulating Turing machines with transformation networks, we prove that it is undecidable whether the strategy will terminate.

Given a Turing machine TM over some alphabet $\Sigma$, we construct a transformation network $N_{\text{TM}} =: ((V, E), T_{\text{TM}})$ and a model assignment $M_{\text{TM},x}$ that are resolvable if, and only if, TM halts on input $x \in \Sigma^*$. We assume that TM contains no self-loops as well as no cycles of length 2, i.e., that each transition and each sequence of two transitions changes the state of TM. This is without loss of generality, since duplication and triplication of each state resolves such self-loops and cycles, respectively. The constructed models consist of a timestamp, the tape content and the tape position (i.e., $\mathbb{M} = \mathbb{N}_0 \times \Sigma^* \times \mathbb{N}_0$). The network $N_{\text{TM}}$ has TM's states as vertices and exactly one directed edge (in arbitrary direction) between each pair of states having a transition between them. The transformations increment the timestamp, change the tape content and update the tape position according to TM's transition if, and only if, the source model's timestamp is higher than the target model's timestamp. More formally, let $\text{Tr}(a, b) \subseteq \Sigma \times \{-1, 0, 1\} \times \Sigma$ be the transitions defined between the states $a$

and $b$ (with $-1$, $0$ and $1$ indicating the head movements "left", "stay" and "right"). We define $T_{\text{TM}}$ with $w|_{p\leftarrow r} := w[0\,..\,p{-}1] \cdot r \cdot w[p{+}1\,..\,|w|{-}1]$ such that:

$$\forall (a,b) \in E : T_{\text{TM}}(a,b)(\alpha =: (t_a, w_a, p_a), \beta =: (t_b, w_b, p_b))$$
$$= \begin{cases} (\alpha, (t_a{+}1,\, w_a|_{p_a\leftarrow r},\, p_a{+}d)) & \text{if } t_a > t_b \wedge \exists\, (w_a[p_a], d, r) \in \text{Tr}(a,b) \\ ((t_b{+}1,\, w_b|_{p_b\leftarrow r},\, p_b{+}d),\, \beta) & \text{if } t_a < t_b \wedge \exists\, (w_b[p_b], d, r) \in \text{Tr}(b,a) \\ (\alpha, \beta) & \text{else} \end{cases}$$

Let $s$ be the initial state of TM. We set

$$M_{\text{TM},x} : v \mapsto \begin{cases} (1, x, 0) & \text{if } v = s \\ (0, \varepsilon, 0) & \text{else} \end{cases}$$

**Lemma 2.** *Executing the transformations of $N_{\text{TM}}$, with initial model assignment $M_{\text{TM},x}$, until no transformations change the model assignment anymore terminates if, and only if,* TM *halts on input $x$. If executing the transformations terminates with the final model assignment $M_f$, then the model with the highest timestamp in* $\text{Im}(M_i)$ *contains* $\text{TM}(x)$ *as tape content.*

*Proof.* We can see by induction over the model assignments $M_i$, $i \in \mathbb{N}_0$ created while executing the transformations:

1. There is exactly one $v \in V$ such that the model $M_i(v) =: (t, x, p)$ has the highest timestamp $t$ of all models in $\text{Im}(M_i)$.
2. There is at most one edge $(a, b) \in E$ whose transformation is inconsistent, i.e., $(M_i(a), M_i(b)) \notin R_{T_{\text{TM}}(a,b)}$. This follows from the definitions of TM and the last executed transformation. Additionally, $a = v$ or $b = v$, because otherwise there would have been two transformations to which models in $\text{Im}(M_{i-1})$ are inconsistent. We assume without loss of generality $a = v$.
3. If $(a, b)$ exists, then $m' := M_{i+1}(b)$ will contain the same tape content and the same tape position as would result if TM was executed one step from state $v$ with tape content $x$ and tape position $p$. Additionally, $m'$ will be the model with the highest timestamp of all models in $\text{Im}(M_{i+1})$.
4. $(a, b)$ does not exist if, and only if, TM would halt in state $v$ with tape content $x$ and tape position $p$. □

**Theorem 2.** *Let $\mathcal{S}$ be an execution strategy that executes transformations until a consistent model assignment is reached. There are inputs for which it can not be decided whether $\mathcal{S}$ will terminate.*

*Proof.* It follows from Lemma 2 that deciding whether $\mathcal{S}$ terminates could decide the halting problem for a universal Turing machine. □

Even worse, this construction makes it unlikely that we will find a practicable criterion that ensures success of an execution strategy like we have motivated in Section 2.4. Because we want the criterion to apply to a single syncx, it would need to restrict the syncx so much that it makes building a network simulating

Turing machines out of the syncx impossible. But since the definition of the syncx in $\mathrm{Im}(T_{\mathrm{TM}})$ is structurally simple, it seems unlikely that a syncx fulfilling the hypothetical criterion would still be apt for most practical use cases.

We could avoid undecidability if we restricted the models' size. The models could then no longer store an unbounded tape and, thus, only simulate space-restricted Turing machines. There is, however, no reasonable bound for a *necessary* model size, to which they could be limited. In consequence, determining a universal space bound for models would be an arbitrary and thus impractical restriction.

Finally, one could question whether it is relevant if an execution strategy can be guaranteed to terminate. Execution strategies will be used to tell users whether changes they made can be incorporated into the other models automatically. In consequence, users should reliably and timely get a response. We might compare this situation to merging changes in version control systems. There, users also want a reliable and timely response on whether their changes could be incorporated automatically, or whether they need to resolve conflicts manually.

## 5 Proposed Strategy

As a consequence of the previous findings, every universal execution strategy will be *conservative*: there will be inputs for which it fails, even though there would have been an execution order leading to a consistent model assignment. In this section, we discuss how to find an appropriate execution order and bound, and finally present the "explanatory strategy", constituting contribution *C3*.

### 5.1 Execution Order: Providing Explainability

Increasing the number of transformation executions an execution strategy permits, lowers its level of conservativeness. In contrast, the effects of different orders in which transformations can be executed are not as easy to categorise. The authors developed a model transformation network simulator [11], whose source code is available at GitHub [10]. It allows to construct transformation networks and to define execution strategies, which can be applied step by step. All examples presented in this paper are also modelled in the simulator. For each examined systematic execution order, such as a depth-first or breadth-first selection, the authors found categories of networks on which the order performed worse than another one in terms of conservativeness. In consequence, conservativeness is not a good sole criterion to evaluate orders by.

We know that a universal execution strategy will inevitably be conservative, i.e., possibly fail for resolvable inputs. In practice, it will be important how well an execution strategy provides explainability in such cases, i.e., helps users to understand where and why the strategy failed with the selected execution order. The order plays a decisive role in this regard, which is why we focus on finding a strategy that improves the order. Imagine, for instance, that the strategy executed transformations in an arbitrary order until some limit is reached. Users might then be confronted with a situation where all transformations have been executed, but the last model assignment is only consistent with some of them. There would be no clear pattern and little clues for users where to start investigating the failure's cause. To improve explainability, the authors thus propose the following principle for an execution order:

**Principle 1.** *Ensure consistency among the transformations that have already been executed before executing a transformation that has not been executed yet.*

Since a syncx can change both models, executing it may results in models that are inconsistent with the syncx that have been executed previously. Following Principle 1, these inconsistencies should be addressed first. In effect, a strategy applying the principle will maintain a subnetwork of syncx with a consistent model assignment and try to expand the subnetwork transformation by transformation.

To exemplify how Principle 1 provides *explainability*, suppose that an execution strategy applying that principle fails after having executed the set of syncx $E \subseteq \mathbb{T}$. Let $\vec{t} \in E$ be the last syncx that was executed for its first time. The strategy can then inform users that integrating $\vec{t}$ into the subnetwork induced by $E$ failed. Furthermore, it can inform users that a result that is consistent with the syncx in $E \setminus \{\vec{t}\}$ exists. By that, users gain valuable information for handling the error: First, when trying to understand the error, they can ignore any syncx that is not in $E$. Second, some aspect of consistency that is present in the consistency relation realised by $\vec{t}$, but absent in the consistency relations realised by the syncx in $E \setminus \{\vec{t}\}$, hinders the strategy from creating a consistent result. Third, when users try to find a consistent model assignment manually, they can start with the consistent result that exists for $E \setminus \{\vec{t}\}$ instead of having to start from scratch.

### 5.2 Execution Bound: Reacting to Each Other

As we have seen, we need to restrict the number of transformation executions with a function in $\omega(m)$ ($m$ being the number of syncx in the input network). Such a limit must be reasonable to support most practical use cases: Not allowing enough transformation executions reduces the usefulness of the strategy since not all useful networks can be resolved. Allowing too many executions might make the strategy run for a long time before aborting, without adding much value.

In Section 4.1, we have motivated that syncx should be able to "react" to each other. We have seen that this excludes any bound in $\mathcal{O}(1)$ for the number of executions per transformation, but to guarantee termination we can also not allow transformations to react to each other indefinitely. If a syncx $\vec{t}$ changes the models and the other already executed syncx have reacted to those changes by adapting the models to be consistent with them as well, $\vec{t}$ should not react by changing the models again. Because if $\vec{t}$ changed the models again, this could easily result in executing the same sequences of transformations repeatedly and there would likely be no consistent result.

We call transformations that behave in the described way *$N$-converging*. This is not a property of a syncx on its own but relative to its network $N$. Thus, it cannot be achieved just by proper construction of an individual transformation.

**Algorithm 1.** The explanatory strategy in pseudocode.

```
Procedure propagate (network, changes):
    executed ← ∅
    accumulatedChanges ← changes
    Invariant: accumulatedChanges applied to network consistent to executed
    while network.contains (candidate | candidate ∉ executed
      ∧ accumulatedChanges.adjacentTo (candidate)) do
        candidateChanges ← candidate.execute (accumulatedChanges)
        subnetwork ← network.edgeInducedSubgraph (executed)
        propagationChanges ←
          propagate (subnetwork, accumulatedChanges ∪ candidateChanges)
        candidateChanges ← candidate.execute (propagationChanges)
        if candidateChanges.adjacentToAny (executed) then
            // Only happens if candidate is not network-converging
            fail (executed, propagationChanges)
        accumulatedChanges ← propagationChanges ∪ candidateChanges
        executed ← executed ∪ candidate
    return accumulatedChanges
```

There is, unfortunately, also no simple way to check it statically. Nevertheless, it captures the sensible expectation for transformations explained above. We yield an execution bound for a strategy by only requiring it not to fail if all syncx are $N$-converging. We will see how this execution bound behaves in combination with Principle 1 in the subsequently presented execution strategy.

**Definition 8.** *Let $N =: (G, T)$ be a transformation network. A syncx $\vec{t} \in \mathrm{Im}(T)$ is* N-converging *if for every initial model assignment and each subset of the syncx $T_p \subseteq \mathrm{Im}(T)$ with $\vec{t} \in T_p$ the resulting model assignment is consistent to $\vec{t}$ whenever $\vec{t}$ has been executed after a sequence of the syncx in $T_p$ that contains each permutation of those syncx as a (not necessarily continuous) subsequence.*

We only require that the sequence of transformation executions contains each permutation, but allow other executions in between. As an example, assume a network $N$ of $N$-converging syncx $\vec{t}_1$, $\vec{t}_2$ and $\vec{t}_3$. After executing them in the order $\vec{t}_1\,\vec{t}_2\,\vec{t}_3\,\vec{t}_1\,\vec{t}_2\,\vec{t}_3$, the current model assignment may still be inconsistent with $\vec{t}_1$ because $\vec{t}_1$ was not executed after the order $\vec{t}_3\,\vec{t}_2$. After executing $\vec{t}_1$ once more, the resulting model assignment must now be consistent with all syncx: $\vec{t}_1$ was executed after the two orders of other syncx $\vec{t}_2\,\vec{t}_3$ and $\vec{t}_3\,\vec{t}_2$. Likewise, $\vec{t}_2$ was executed after $\vec{t}_1\,\vec{t}_3$ and $\vec{t}_3\,\vec{t}_1$, and $\vec{t}_3$ was executed after $\vec{t}_1\,\vec{t}_2$ and $\vec{t}_2\,\vec{t}_1$.

### 5.3 The Explanatory Strategy

We now turn to a concrete strategy that realises the discussed design choices. Algorithm 1 gives pseudocode for such a strategy, which we call the "explanatory

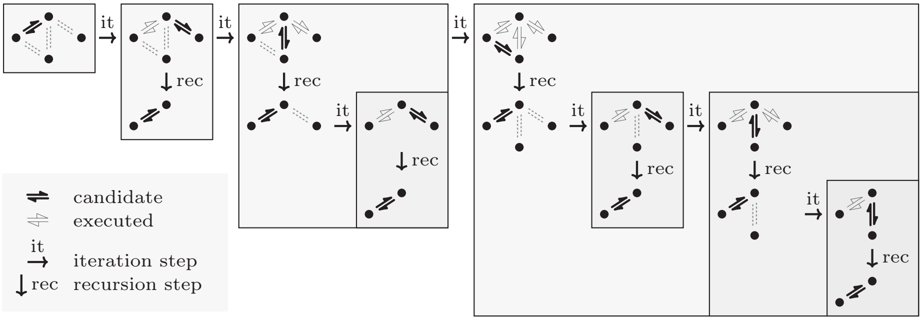

**Fig. 4.** Exemplary execution of the explanatory strategy for a change in the topmost model, depicting the iterations (horizontal) and recursion steps (vertical).

strategy". At a high level, it acts like this: Given a changed model assignment, the strategy picks the next `candidate` syncx to execute. After executing the candidate, the strategy calls itself on the `subnetwork` formed by the already executed syncx. By that, it propagates the changes of the last execution throughout the `subnetwork` and ensures that they are consistent with the `executed` syncx. Finally, the strategy executes the initial candidate again to ensure that the changes added during the subnetwork propagation are consistent with the `candidate`. If that repeated execution of the `candidate` generates new changes in any model that is kept consistent by an already executed syncx, the execution fails, because the `candidate` does not fulfil the definition of being $N$*-converging*, as we will see in the following. In that case, the procedure returns the already `executed` syncx to which consistency was restored by the also returned changes in order to support a user in examining the reasons for the strategy to fail. If the models are consistent with the `candidate`, the strategy picks the next one. In effect, the strategy realises Principle 1 in a recursive fashion and ensures that each permutation of all yet executed syncx is executed at every recursion level.

Figure 4 depicts an exemplary execution of the strategy for a network with four models and four transformations. We assume that after an initially consistent state of the models, the topmost one was modified. We can see that each recursion only treats the `subnetwork` of previously executed transformations. Hence, the `network` gets smaller at each recursion level.

Unlike the formalisation in Section 2.3, the presented algorithm is based on changes instead of model states. Changes contain information that cannot be recovered by comparing model states [6]. Thus in practice, we want to support change-based execution. The algorithm also uses changes to determine potential candidates for the next transformation to execute: It only picks candidates that are adjacent to a model that was changed. The input `changes` describe all changes that occurred since the last model assignment $M$ that was known to be consistent. The procedure returns `accumulatedChanges` that, when applied to $M$, yield a new model assignment $M'$. For our formalisation, $M'$ is the algorithm's output.

We discuss some implementation details for the explanatory strategy further below. First, we prove that the strategy has indeed the motivated properties. We assert that it terminates always and determine its execution bound.

**Theorem 3.** *The explanatory strategy terminates for every input.*

*Proof.* Because all called functions terminate, only the loop (Line 5) and the recursive call in Line 8 can lead to non-termination. Let $m$ denote the number of edges of `network`. The set `executed` is initialised to be empty (Line 2) and grows by one element in every iteration of the loop. The loop is executed no more than $m$ times, because after $m$ iterations there is no transformation that is not in `executed` and, thus, the loop condition cannot be fulfilled.

The recursive call receives a network that is smaller than `network` in terms of edges, because it does not contain the current `candidate`. If `network` is empty, then the algorithm will not enter the loop and not make a recursive call. Hence, the recursive stack never gets higher than $m$. □

**Theorem 4.** *The explanatory strategy executes syncx at most $\mathcal{O}(2^m)$ times.*

*Proof.* Let $T(m)$ denote the number of syncx executions the algorithm invokes for a `network` with $m$ edges. The set `executed` is initialised to be empty and grows by one syncx every loop iteration (Line 13). It follows that the recursive call in Line 8 receives a network that is one syncx larger each time. Thus, we find

$$T(0) = 0,\; T(m) = 2m + \sum_{i=0}^{m-1} T(i) = 2 + 2\,T(m-1) = 2\,(2^m - 1) \in \mathcal{O}(2^m) \;\square$$

Next, we show that the strategy fulfils the fundamental Requirements 1 and 2 regarding correctness and hippocraticness, which we defined in Section 2.4.

**Theorem 5.** *The explanatory strategy is correct.*

*Proof.* Assume the contrary, i.e., that the strategy produces a model assignment $M$ for network $N$ such that $M \notin R_N$. That means that there is an edge $(a, b) \in E$ such that $(M(a), M(b)) \notin R_{\vec{t}}$, where $\vec{t} := T(a, b)$. We distinguish these cases:

1. $\vec{t}$ was never executed. Then `accumulatedChanges` never contained any change adjacent to $a$ or $b$ (Line 5). Since the initial `changes` were relative to a consistent model assignment, we know that $(M(a), M(b)) \in R_{\vec{t}}$.
2. $\vec{t}$ was executed and no other transformation adjacent to $a$ or $b$ was executed afterwards. Then $(M(a), M(b)) \in R_{\vec{t}}$ per definition.
3. $\vec{t}$ was executed and another transformation $\vec{u}$ adjacent to $a$ or $b$ was executed afterwards. Because $\vec{u}$ was executed after $\vec{t}$, $\vec{t}$ was in `executed` when $\vec{u}$ was the `candidate`. So $\vec{t}$'s last execution was in the recursion after $\vec{u}$'s first execution in Line 6. Afterwards, $\vec{u}$ was only executed in Line 9. If $\vec{u}$ would have changed $M(a)$ or $M(b)$, the strategy would have raised a failure. Hence, $M(a)$ and $M(b)$ are the same as after the execution of $\vec{t}$, and $(M(a), M(b)) \in R_{\vec{t}}$.

All cases lead to a contradiction. □

**Theorem 6.** *The explanatory strategy is hippocratic.*

*Proof.* The strategy only produces changes by executing syncx, which, per definition, only generate changes if the models are not in their consistency relations. □

Finally, we verify that we have indeed realised Principle 1 and that the strategy does not fail for a network $N$ of only $N$-converging transformations.

**Theorem 7.** *The explanatory strategy ensures consistency among the transformations that have already been executed before executing a transformation that has not been executed yet (see Principle 1).*

*Proof.* After the recursive call in Line 8, the current model assignment is consistent with all `executed` syncx (Theorem 5) and no changes to models adjacent to an executed syncx are allowed. □

**Theorem 8.** *If the input* `network` *of the explanatory strategy consists only of* `network`*-converging syncx, then the explanatory strategy does not fail.*

*Proof.* First, we note that when calling the algorithm on a `network` with $m$ transformations, the first $m-1$ iterations of the loop act identically to executing the algorithm on a network without the last `candidate`. Second, we note that the second part of the loop condition, "`accumulatedChanges`.*adjacentTo* (`candidate`)" (Line 5), does not change the algorithm's result apart from controlling the order in which the syncx are executed. If any syncx was never executed because of this condition, then executing it would not have changed any model. Hence, we assume w.l.o.g. that all syncx in `network` will get executed.

Now we show the following, stronger statement by induction over the number $m$ of edges in `network`: "After running the explanatory strategy, the sequence of executed syncx contains each permutation of those syncx (not necessarily continuously)". Since the transformations are `network`-converging and because of our first note above, proving this statement shows that the condition leading to a failure (Line 10) will never evaluate to true. The statement is trivially true for $m = 1$. Assume that the statement is true for all networks of size $1 \leq n < m$ but not true for a network of size $m$. That means that after executing the last iteration of the loop, there is an order $o$ of the $m$ syncx in `network` in which they have not been executed yet. Let $\vec{t}$ be the `candidate` of the last iteration. Let $j$ be the index of $\vec{t}$ in $o$. Per induction assumption, the order $o[1] \ldots o[j-1]$ has been executed in the previous iterations of the loop. Afterwards, $\vec{t}$ was executed in Line 6. Per induction assumption, the order $o[j+1] \ldots o[m]$ has been executed in the recursive call (Line 8) of the last iteration. This happened after Line 6. Hence, the transformations have been executed in the order $o$. This is a contradiction. □

The explanatory strategy only guarantees to produce a consistent model assignment if all syncx are $N$-converging. We can, unfortunately, not provide an approach to achieve $N$-convergence by construction or to determine $N$-convergence. We have, however, also discussed that every universal execution strategy needs to operate conservatively and thus fails in certain cases. Thus, even if a network $N$

contains syncx that are not $N$-converging, the explanatory strategy still operates conservatively and at least fails based on the notion of a sensible and well-defined property. In addition, the exponential worst-case performance of the strategy is no limitation, because it does only represent a bound to ensure termination. In cases in which the strategy terminates, we expect the repeated execution of each syncx to perform only few changes in reaction to the changes made by other syncx, as otherwise they are unlikely to be $N$-converging. The interested reader can try out the explanatory strategy using the previously mentioned simulator [11].

In its current formulation, the explanatory strategy does not prevent the syncx from overwriting the initial user changes. This seems inappropriate, as user changes should usually not be reverted. Other authors address this issue by forbidding changes to models that have been edited by users [3, 30, 29], called "authoritative models". There are, however, practical use cases where such changes should be allowed—the example in Section 4.1 is one of them. An option would be to let the strategy fail as soon as a syncx execution overwrites a user change.

## 6 Conclusion

In this paper, we have discussed influencing factors for designing a universal execution strategy for model transformation networks. Such a strategy orchestrates transformations to create a consistent set of models. It involves determining an order to execute the transformations in, and a bound for the number of executions. We have proven that every universal execution strategy that always terminates needs to be conservative, i.e., it will fail for certain cases in which an execution order of transformations that yields a consistent solution exists. We have argued that providing explainability in cases where an execution strategy fails should be a central design goal. As a result, we have proposed the *explanatory strategy*, which is proven correct and terminates for every input. Additionally, it improves explainability of failures and has a well-defined bound for the number of transformation executions to ensure a reasonable level of conservativeness.

We have formalised our findings on execution bounds and the behaviour of the proposed execution strategy to prove the insights and expected properties of the strategy. In consequence, this paper provides fundamental knowledge about the design space and relevant design goals of transformation network execution strategies. While the statements on correctness and well-definedness are proven, those on the usefulness of the strategy were derived by argumentation. To improve evidence of the results, the authors plan to apply the strategy to realistic use cases, involving larger networks of more complex transformations.

Furthermore, the authors want to examine how the strategy can be further optimised: It might, e.g., be improved by backtracking and trying further candidate transformations, or by selecting the next candidate more carefully. Since early executed transformations will be executed most often, starting with those that will most unlikely cause conflicts might be beneficial. Finally, this paper assumes transformations to be binary. Since the presented strategy does not require this, future research could investigate transferability to multiary transformations.

## Image Sources paintingred: "Default Avatar Headshot Icons", found on Vecteezy.
https://www.vecteezy.com/vector-art/141712-default-avatar-headshot-icons.
Vecteezy Free License.

Object Management Group: UML logo.
https://www.uml.org/index.htm.
Trademark.

Palladio logo.
https://sdqweb.ipd.kit.edu/wiki/File:Palladio-Logo-stilisiert-vektor.pdf.
Authorized use.

The Linux Foundation: OpenAPI™ logo.
https://github.com/OAI/OpenAPI-Style-Guide/blob/master/graphics/vector/OpenAPI_Logo_Black.svg. Trademark.

Freepik: "Computer".
https://www.flaticon.com/free-icon/computer_1077701.
Flaticon Basic License.

**Open Access** This chapter is licensed under the terms of the Creative Commons Attribution 4.0 International License (http://creativecommons.org/licenses/by/4.0/), which permits use, sharing, adaptation, distribution and reproduction in any medium or format, as long as you give appropriate credit to the original author(s) and the source, provide a link to the Creative Commons license and indicate if changes were made.

The images or other third party material in this chapter are included in the chapter's Creative Commons license, unless indicated otherwise in a credit line to the material. If material is not included in the chapter's Creative Commons license and your intended use is not permitted by statutory regulation or exceeds the permitted use, you will need to obtain permission directly from the copyright holder.
